# Exhaustive Exercise Attenuates the Neurovascular Coupling by Blunting the Pressor Response to Visual Stimulation

**DOI:** 10.1155/2015/671063

**Published:** 2015-03-19

**Authors:** Yuji Yamaguchi, Tsukasa Ikemura, Naoyuki Hayashi

**Affiliations:** ^1^Graduate School of Human-Environment Studies, Kyushu University, Kasuga, Fukuoka 816-8580, Japan; ^2^Graduate School of Decision Science and Technology, Tokyo Institute of Technology, Meguro, Tokyo 152-8852, Japan; ^3^Faculty of Sports Sciences, Waseda University, Tokorozawa, Saitama 359-1192, Japan

## Abstract

Neurovascular coupling (NVC) is assessed as an increase response to visual stimulation, and is monitored by blood flow of the posterior cerebral artery (PCA). To investigate whether exhaustive exercise modifies NVC, and more specifically, the relative contributions of vasodilatation in the downstream of PCA and the pressor response on NVC, we measured blood flow velocity in the PCA (PCAv) in 13 males using transcranial Doppler ultrasound flowmetry during a leg-cycle exercise at 75% of maximal heart rate until exhaustion. NVC was estimated as the relative change in PCAv from the mean value obtained during 20-s with the eyes closed to the peak value obtained during 40-s of visual stimulation involving looking at a reversed checkerboard. Conductance index (CI) was calculated by dividing PCAv by mean arterial pressure (MAP) to evaluate the vasodilatation. At exhaustion, PCAv was significantly decreased relative to baseline measurements, and the PCAv response to visual stimulation significantly decreased. Compared to baseline, exhaustive exercise significantly suppressed the increase in MAP to visual stimulation, while the CI response did not significantly change by the exercise. These results suggest that exhaustive exercise attenuates the magnitude of NVC by blunting the pressor response to visual stimulation.

## 1. Introduction

Neurovascular coupling (NVC) is important to maintain adequate blood flow to working cerebral regions when information processing occurs. NVC has been assessed as the regulation of blood flow to the visual cortex by measuring the increase in blood flow of the posterior cerebral artery (PCA) in response to a given visual stimulation, as evaluated using transcranial Doppler (TCD) ultrasound [1]. PCA is the main artery that supplies blood to the visual cortex, and an increase response in PCAv to visual stimulation has been believed to be induced by regional vasodilatation accompanied with the underlying cortical neural activity [2]. Impaired NVC in the PCA should be avoided to ensure adequate blood flow in working regions.

There is evidence in the literature that suggests that NVC is altered during or by certain physiological conditions. For instance, NVC was reportedly maintained during aerobic exercise at 70% of maximal heart rate (HR) [3], at various intensities of submaximal dynamic exercise [4], as well as during static exercise [5], hypercapnia [6], and orthostatic stress [7], and increased during the cold pressor test [8]. However, previous studies revealed that there was a decrease in NVC during hyperventilation-induced hypocapnia at rest [9]. NVC should be maintained during exhaustive exercise because it is important to support a stable or increased metabolism in the visual cortex against a decrease in the global cerebral blood flow (CBF) due to lower *P*_a_CO_2_ via hyperventilation-induced cerebral vasoconstriction [10]. Considering that exhaustive exercise has been associated with a hypocapnic environment, it is interesting to inquire how exhaustive exercise affects NVC. While it has been illustrated that NVC is maintained during submaximal exercise, it is currently unknown how exhaustive exercise affects NVC.

We hypothesized that NVC might be altered during exhaustive exercise because of two factors related to possible mechanisms of NVC: hypocapnia and a changed mean arterial pressure (MAP). Hypocapnia, which occurs during high-intensity exercise, induces vasoconstriction in the cerebral vessels, decreasing blood flow velocity in the PCA (PCAv), and consequently inhibits NVC [9].

A change in MAP during exhaustive exercise is another important factor that may decrease NVC. Our previous study indicated that the pressor response to visual stimulation plays a role in NVC, as well as local vasodilatation at rest and during static exercise, and that an increase in the pressor response to visual stimulation preserved NVC against a blunted vasodilatory response during static handgrip exercise at 30% maximum voluntary contraction [5]. It was suggested that NVC is maintained during exercises via contributions by both the pressor response and vasodilatation, calculated from PCAv and MAP, in response to visual stimulation. Thus, it is uncertain which of these two factors has a greater impact on NVC.

The purpose of the present study was to investigate the effects of the pressor and regional vasodilatory responses to visual stimulation on NVC during exhaustive exercise. In this study, we measured PCAv and pressor responses to visual stimulation during a leg-cycle exercise at 75% of maximal HR, which was performed until exhaustion. We previously found a blunting of the pressor response to visual stimulation during high-intensity dynamic exercise [4]. Based on this data, we hypothesized that exhaustive exercise would reduce baseline PCAv and attenuate the magnitude of the response in the PCAv to visual stimulation. In addition, we aimed to confirm the role of the MAP on NVC.

## 2. Materials and Methods

### 2.1. Subjects

Thirteen healthy, active, nonsedentary males (age 21 ± 0.8 years (mean ± SE), height 173 ± 1 cm, body mass 64 ± 2 kg) participated in this study. All of the subjects were nonsmokers, normotensive, and free from any known autonomic dysfunction and cardiovascular disease and were not taking any medications. The Ethics Committee of the Institute of Health Science, Kyushu University, Japan, approved the experimental protocol, and all subjects provided written informed consent to participate prior to the commencement of the study. Every protocol used conformed to the Declaration of Helsinki. Each subject visited the laboratory before taking part in the experiments for familiarization with the techniques and procedures of the protocol. Subjects who normally wore glasses took them off before the experiment.

### 2.2. Protocol

The subjects arrived at the laboratory after having abstained from caffeinated beverages and strenuous exercise for 6 h, and from eating for at least 2 h before the experiment; they had not experienced sleep loss the previous night. All studies were performed in a darkened and quiet room at an ambient temperature of 22°C. The individual target work rate at 75% of their maximal heart rate (136 ± 6 W) was determined using an incremental cycle ergometer test at least seven days prior to the experiment. After a 2 min resting period, the subjects began cycling at half of the target work rate. After 1 minute, the exercise intensity was increased to the target work rate. This exhaustive exercise was continued until subjects could no longer maintain a pedaling cadence of 60 rpm. Following the exercise test, subjects pedaled at 20 W for cooling down for 1 minute prior to a 2 min recovery period. Subjects received visual stimulation for 2 min at resting baseline and recovery and every 5 min after the onset of exercise until exhaustion.

### 2.3. Measurements

Blood pressure (BP), HR, and PCAv were continuously recorded throughout the trial. End-tidal carbon dioxide partial pressure (*P*_ET_CO_2_) and NVC data were obtained at resting and recovery and every 5 min during exercise. External ear temperature was recorded before and after the exhaustive exercise.

Beat-by-beat BP was monitored with a continuous finger photoplethysmography device attached to the left middle finger (Finometer, Finapres Medical Systems, Amsterdam, The Netherlands). HR was measured continuously using a standard electrocardiogram (ECG; MEG2100, Nihon-Kohden, Tokyo, Japan). These analogue signals were sampled at 1 kHz using an A/D converter (PowerLab 8/30, ADInstruments, Colorado Springs, CO, USA). Minute-by-minute HR and MAP were calculated from the ECG and BP recordings. *P*_ET_CO_2_ and tidal volume were monitored with a gas analyzer (AE-310 s, Minato Medical Science, Tokyo, Japan), and the *P*_a_CO_2_ was estimated [11]. External ear temperature was measured using an infrared ear thermometer (MC-510, Omron Healthcare, Kyoto, Japan), which detects the tympanic temperature with infrared rays irradiated toward the tympanum from the entrance to the ear canal.

Mean PCAv was obtained by transcranial ultrasonography (WAKI, Atys Medical, St-Genis-Laval, France). A 2 MHz Doppler probe was placed at the right temporal window and fixed using a headband. The vessel was identified by TCD ultrasonography according to standard criteria [12]. Middle cerebral artery (MCA) was insonated at a depth of 50–60 mm with a standard hand probe, followed by insonation of the PCA at a depth of 60–70 mm. To confirm the accurate insonation of the PCA, we performed ipsilateral carotid compression, which increased PCAv and decreased MCAv [13]. The length of the sample volume was set at 6 mm.

### 2.4. Visual Stimulation

The visual stimulus was comprised of two repetitions: a 20 s eyes closed and 40 s visual stimulation period, using a reversed checkerboard. Subjects were seated 0.5 m from the front of a 24-inch flat computer screen (visual angle of 25°). During the stimulation period, subjects were asked to gaze at a small red spot at the center of the computer screen. During the eyes closed period, the screen was masked and the subjects were asked to close their eyes. The checkerboard pattern comprised of black and white squares arranged with a spatial frequency of 1.6 cycles/degree. The black-and-white squares were alternated at a frequency of 2 Hz.

### 2.5. Data Analysis

We estimated NVC as the relative change in the PCAv between the mean value during the 20 s with the eyes closed and the peak response during the 40 s visual stimulus. PCAv response to visual stimulation was averaged over two repetitions for each individual. The peak velocity in response to visual stimulation was identified, and this was averaged across two repetitions. Conductance index (CI) of the cerebral vessel was calculated by dividing PCAv by MAP at the corresponding time points.

The data were expressed as the mean ± SE values. The effects of time on PCAv, MAP, and CI responses to visual stimulation were examined by a one-way repeated-measures ANOVA. When a significant *F* value was detected, this was analyzed further against the baseline value using Bonferroni's post hoc test. HR, MAP, *P*_a_CO_2_, external ear temperature, PCAv, and CI of the nonstimulation (eyes closed) period during exercise were compared to their resting baseline counterparts using a paired *t*-test. PCAv, MAP, and CI responses to visual stimulation were compared to the prestimulation (eyes closed) baseline data using a paired *t*-test. The level of statistical significance was set at *P* < 0.05. All of the statistical analyses were performed with the SPSS software program (PASW statistics 18, SPSS, IL, USA).

## 3. Results

### 3.1. Systemic Changes

**Figure 1:**
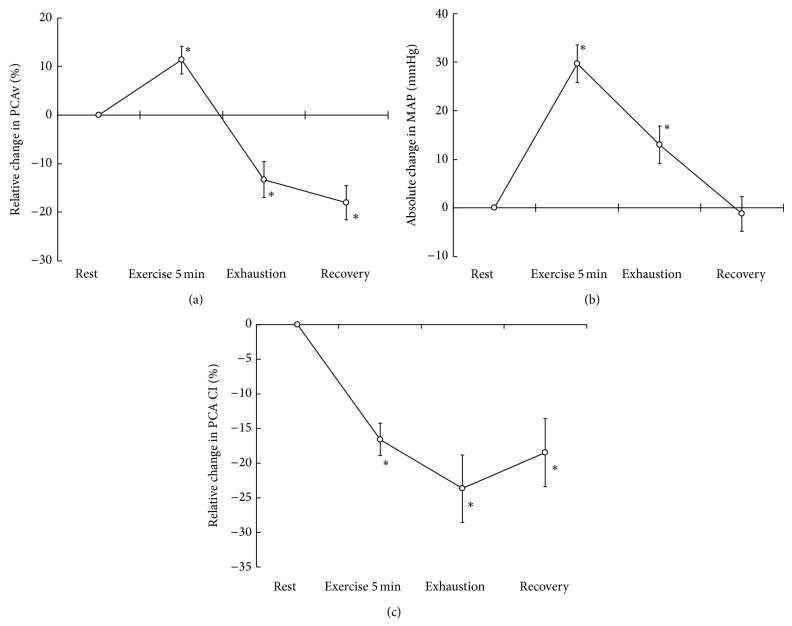
The relative changes in the blood flow velocity of the posterior cerebral artery (PCAv) (a), the absolute changes in the mean arterial pressure (MAP) (b), and the relative changes in the conductance index (CI) (c) from the resting baseline to the exercise and recovery periods. ^*^ *P* < 0.05 versus resting baseline.

The duration to exhaustion was 23 ± 2 min (range 13–30 min). HR significantly increased above baseline levels throughout the exercise session (*P* < 0.05; Table 1). Exercise significantly increased the MAP, while it returned to baseline level after the cessation of exercise (Table 1, Figure 1(b)). *P*_a_CO_2_ significantly increased at 5 min after the onset of exercise, but significantly decreased at exhaustion and during the recovery period (−7 ± 2 and −11 ± 1%, resp., *P* < 0.05). External ear temperature significantly increased after the exhaustive exercise from baseline (35.8 ± 0.1 and 36.6 ± 0.2°C, resp., *P* < 0.05).

### 3.2. Prestimulation PCAv during Exhaustive Exercise

Five minutes after the beginning of the exercise, PCAv significantly increased from baseline (11.3 ± 2.8%, *P* < 0.05; Table 1; Figure 1(a)). At exhaustion, it significantly decreased by −13.3 ± 3.7% from baseline and continued to decrease during the recovery period (−18.0 ± 3.5%, *P* < 0.05). CI in the PCA was significantly decreased at 5 min after the beginning of exercise, at exhaustion, and during the recovery period (−16.6 ± 2.4, −23.7 ± 4.9, and −18.5 ± 4.9%, resp.; Figure 1(c)).

### 3.3. PCAv Response to Visual Stimulation (NVC)

**Figure 2:**
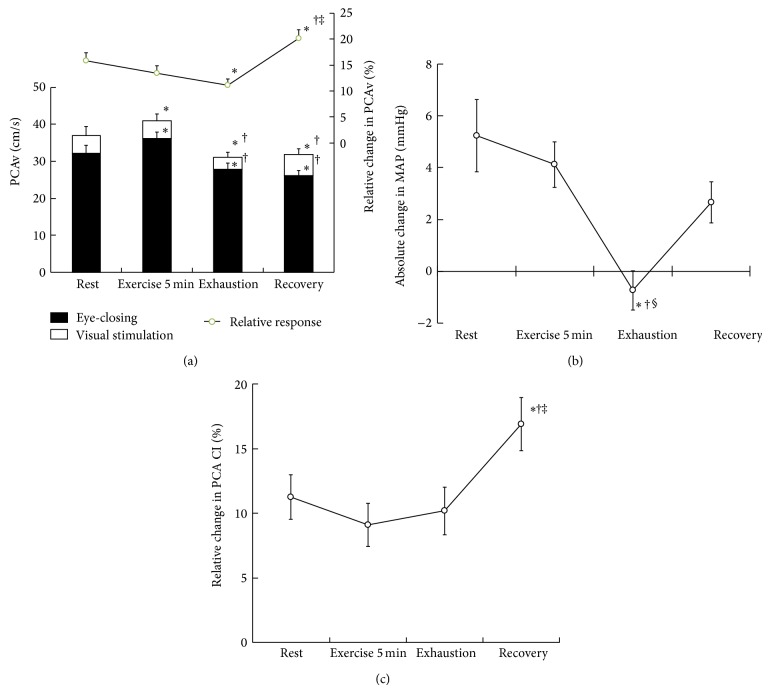
The PCAv during the eyes closed baseline (solid bars) and the increases by the visual stimulation (open bars) and the relative changes in the PCAv (solid line) (a), the absolute changes in the MAP (b), and the relative changes in the CI in response to visual stimulation (c). ^*^ *P* < 0.05 versus resting baseline; ^†^ *P* < 0.05 versus 5 min of exercise; ^‡^ *P* < 0.05 versus exhaustion; ^§^ *P* < 0.05 versus recovery.

NVC, that is, the increase response in PCAv to visual stimulation, was significantly decreased at exhaustion (11.1 ± 1.3%) compared to baseline (15.9 ± 1.5%, *P* < 0.05; Figure 2(a)), whereas it was significantly increased during the recovery period (20.2 ± 1.7%, *P* < 0.05). Conversely, the absolute peak value of PCAv in response to visual stimulation was the same level during the exhaustion and recovery periods (*P* > 0.05; Figure 2(a)). Compared to baseline, exhaustive exercise significantly suppressed the increase response in MAP with visual stimulation from 5.2 ± 1.4 to −0.7 ± 0.8 mmHg (*P* < 0.05; Figure 2(b)), while CI response significantly increased during the recovery period from 11.3 ± 1.7 to 16.9 ± 2.1% (*P* < 0.05; Figure 2(c)).

## 4. Discussion

The main finding of the present study was that exhaustive exercise attenuated the magnitude of NVC by suppressing the pressor response, without any significant change in the vasodilatory response to visual stimulation. In turn, NVC increased after cessation of the exercise, with an enhanced CI response to visual stimulation. Compared with baseline, the prestimulation PCAv was significantly decreased at exhaustion by cerebral vasoconstriction despite the pressor response to exercise, similar to the blood flow response in the MCA [10].

### 4.1. Prestimulation PCAv during Exhaustive Exercise

In the present study, at exhaustion, PCAv was lower than the resting baseline by 13%, and this was accompanied by a decrease in CI of the PCA. *P*_a_CO_2_ decreased by 7% during exhaustive exercise. These findings indicate that the decrease in PCAv was due to hyperventilation-induced cerebral vasoconstriction in the PCA. Regional vasoconstriction in the PCA may have blunted the effect of an enhanced MAP during exhaustive exercise relative to baseline level. The present results support findings by Ogoh et al. [10] who reported reduced blood flow of MCA and *P*_a_CO_2_ during exhaustive exercise.

The decreases in the prestimulation PCAv and CI of the PCA persisted during the recovery period. However, MAP returned to the resting baseline level. Thus, the cerebral vasoconstriction in the PCA must have decreased PCAv. The vasoconstriction may have been induced by the decrease in the *P*_a_CO_2_ after exercise.

Submaximal dynamic exercise would increase the CBF mainly by the pressor response with a lack of vasodilatation. In the present study, the prestimulation PCAv was significantly increased at 5 min after the beginning of exercise in spite of a decrease in the CI. The elevation in the MAP apparently overwhelmed the effects of the decrease in the CI. These results are consistent with our previous study indicating that an increase in the prestimulation PCAv was induced by an elevated MAP, but not by a change in the CI of the PCA, during high-intensity exercise [4].

The increase in the PCAv at 5 min after the beginning of exercise was not explained by hypercapnia-induced vasodilatation in the present study. Indeed, *P*_a_CO_2_ significantly increased from the resting baseline level to 5 min after beginning the exercise. Nevertheless, CI of the PCA decreased, indicating that there was vasoconstriction. Although a regional difference among the various cerebral arteries during dynamic exercise was identified in a previous study, PCA blood flow responded similarly to MCA blood flow during exercise [14]. These results imply that hypercapnia-induced vasodilatation may be less effective for inducing a CBF response than the exercise-induced pressor response during submaximal dynamic exercise. What impact these factors have on PCA blood flow still remains to be determined [15].

### 4.2. PCAv Response to Visual Stimulation (NVC)

NVC, evaluated as an increase response in the PCAv to a given visual stimulation, was attenuated at exhaustion in the present study. This attenuation was due to suppression in the MAP response to visual stimulation, whereas the increase response in the CI of the PCA to the stimulation was not changed at exhaustion compared to the baseline. This trend is consistent with our previous study indicating a limited contribution of the pressor response to NVC during high-intensity dynamic exercise [4]. The present results suggest that exhaustive exercise has an inhibitory effect on NVC, which is accompanied by a suppression of the pressor response to visual stimulation.

An increase in the eyes closed baseline PCAv during submaximal exercise would not be directly related to NVC, as was shown during static exercise [5]. In the present study, the magnitude of NVC was maintained at 5 min after the beginning of exercise, although PCAv when the subjects had their eyes closed was increased by the exercise-induced pressor response. Increases in both the MAP and CI of the PCA in response to visual stimulation were observed, in agreement with our previous study reporting the effect of submaximal dynamic exercise [4].

In the present study, the elevation of MAP associated with visual stimulation contributed to an increase in the PCAv, except for at exhaustion, supporting the contribution of the MAP to NVC. In the classic concept, a regulation of blood flow in the working cerebral regions is mainly achieved by regional vasodilatation associated with the local metabolic demand in the brain [2]. Conversely, we previously suggested that the pressor response to visual stimulation also had a role for accomplishing NVC [4, 5]. The present study supports our previous results.

On the other hand, the role of an elevated MAP on the increase in the PCAv induced by visual stimulation was abolished at exhaustion, in spite of a maintained increase in the CI of the PCA. External ear temperature was increased after the exhaustive exercise. The suppression of the pressor response to visual stimulation at exhaustion may be due to hyperthermia-induced peripheral vasodilatation to redistribute the blood flow to the skin, as reported in previous studies [16–18].

Cerebral vasoconstriction with hyperventilation-induced hypocapnia during exhaustive exercise attenuated NVC, in line with a previous investigation showing an inhibition of NVC in response to visual stimulation during hyperventilation-induced hypocapnia at rest [9]. Based on the present results, *P*_a_CO_2_ was significantly decreased at exhaustion, which consequently attenuated the magnitude of NVC, with a decrease in the prestimulation PCAv. Conversely, according to this finding, NVC was not inhibited although the reduction in the *P*_a_CO_2_ continued during the recovery period. The increased vasodilatory response to visual stimulation overwhelmed the effect of the decreased prestimulation PCAv related to cerebral vasoconstriction in order to preserve NVC during the recovery.

NVC increased after exhaustive exercise, whereas PCAv with the eyes closed decreased relative to baseline level at recovery. This increase in NVC can be explained by the increased CI response, because the regional vasodilation increased. On the other hand, we have no clear explanation for the stable peak value and decreased baseline. It is possible that, after exhaustive exercise, the baseline metabolic demand might decrease according to the decreased ability or demand for visual processing. In turn, once a visual stimulus is given, the metabolic demand might increase as needed, consequently leading to exaggerated vasodilatation to compensate for the decreased baseline to increase the blood flow to a stable peak.

### 4.3. Technical Considerations

TCD ultrasound flowmetry has a potential limitation. This technique is based on a premise condition that a diameter of the target cerebral blood vessel is being constant. Conversely, physiological stimuli reportedly have no effect on the diameter of MCA [19]. Although there is no available data demonstrating that a diameter of PCA kept relatively unchanged during any disturbances, a change in PCAv by means of TCD ultrasonography reflected its blood flow volume.

## 5. Conclusions

Exhaustive exercise decreases the prestimulation PCAv and attenuates the magnitude of NVC. The inhibition of NVC at exhaustion would be mainly due to the suppression of the pressor response to visual stimulation. The present findings support our original hypothesis that the contributions of both pressor and vasodilatory responses to visual stimulation play a role in maintaining the NVC at rest and during exercise.

## Figures and Tables

**Table 1:** The heart rate (HR), mean arterial pressure (MAP), arterial partial pressure of carbon dioxide (*P*_a_CO_2_), and the posterior cerebral artery blood flow velocity (PCAv) during the rest, exercise, and recovery periods.

	Rest	Exercise period		Recovery
		5 min	Exhaustion	
HR (bpm)	69.9 ± 2.5	151.9 ± 4.3^*^	173.4 ± 4.4^*^	117.8 ± 3.5^*^
MAP (mmHg)	87.5 ± 4.0	112.9 ± 5.6^*^	98.3 ± 4.9^*^	85.4 ± 3.6
*P*_a_CO_2_ (mmHg)	37.2 ± 1.1	42.0 ± 1.1^*^	34.3 ± 0.9^*^	32.9 ± 1.1^*^
PCAv (cm/s)	32.2 ± 1.9	36.2 ± 2.6^*^	27.9 ± 2.1^*^	26.1 ± 1.5^*^

The data are presented as the mean ± SE values, ^*^ *P* < 0.05 versus rest.
